# Gynæcology and Obstetrics

**Published:** 1897-06

**Authors:** Walter C. Swayne


### GYNÆCOLOGY AND OBSTETRICS.

The etiology of **displacements of the uterus** which call for operation has been fully dealt with in recent papers by Howard Kelly[1] and Hunter Robb.[2]

Of the operations which have been devised for the cure of retroposition that of **ventrofixation** calls for most comment.

Dührssen[3] considers that the complications following ventrofixation and vaginal fixation can be avoided by **closing the peritoneum separately.** He describes a method of vaginal or rather vesico-uterine fixation. Anterior colpotomy is performed, by transverse incision of the vaginal wall, and longitudinal (median) incision of the utero-vesical fold; the fundus is then seized with bullet forceps and sutured to the vesical peritoneum. Two sutures are used on either side, the upper one being inserted a quarter of an inch above the upper end of the peritoneal opening. These sutures unite vesical and uterine peritoneum, obliterating, to a great extent, the previously existing vesico-uterine pouch. The peritoneal wound is then closed with a continuous catgut suture, care being taken to unite peritoneal tissues only. The vaginal wound is sutured separately in the same manner. The object of this method is to secure peritoneal adhesion only, so that the mobility of the uterus shall be unimpaired within normal limits. The result appears to be as expected in Dührssen's cases, which up to now are too few to allow of a definite opinion being arrived at.

One important point appears to be the **position of the sutures.** Mangiagalli, Sinclair, and Laroyenne suture the middle section of the anterior uterine wall, leaving the fundus free to expand should pregnancy occur. Dührssen places the sutures higher and nearer the fundus, Mackenrodt lower in the inferior segment. The former causes interference with the expansion of the uterus, the latter does not always prevent return of the displacement; the method of Mangiagalli would appear more suitable.

[1] *Am. J. M. Sc.*, 1896, cxii. 629.

[2] *Columbus M. J.*, 1896, xvii. 311; abstract in *Brit. M. J.*, 1896, ii. Epitome, p. 79.

[3] *Centralbl. f. Gynäk.*, 1896, xx. 584; abstract in *Am. J. M. Sc.*, 1896, cxii. 622.

Kelly[1] prefers **ventrofixation.** After freeing adhesions, he sutures the fundus to the abdominal wall by two stitches passed a little below the fundus on its posterior aspect. He uses silk sutures and leaves them permanently in position. In one case, in which he obtained the uterus after *post-mortem* examination, two thin bands of adhesions were found uniting the posterior aspect of the fundus to the abdominal wall; the *post-mortem* specimen was obtained over a year after the operation. He fixes the uterus by passing two silk sutures through the fundus uteri and the peritoneum and sub-peritoneal tissue of the abdominal wall. One is passed through the tissue of the fundus about $\frac{1}{2}$ inch from its summit and on its posterior surface, the other about $\frac{1}{3}$ to $\frac{1}{2}$ an inch lower. The upper uterine suture is attached to the wall of the abdomen at a lower point than the lower uterine suture, so that the uterus is held in a position of anteversion and flexion. Silk is the material used, and the sutures are buried. The wound is then closed in the ordinary way. He reports 219 cases operated on by him; no death occurred. In fourteen cases pregnancy subsequently took place. In one case delivery was difficult, owing to dense adhesions, the result of suppuration, having occurred; the labour was terminated by the use of forceps.

Küstner[2] reports 1120 cases of ventrofixation: in 637 the retroversion was adherent; in 442 non-adherent. Seven deaths occurred, two of which were due to intestinal obstruction. Forty-four failures occurred. Pregnancy was recorded in 122 cases: in seventy-four pregnancy and parturition were normal; in fifteen abortion or miscarriage occurred. Transverse presentations occurred three times; tubal pregnancy once; retention of placenta once, and in three cases Cæsarean section was necessary.

It will thus be seen that the **mortality after ventrofixation** is slightly higher than that after vaginal fixation; but in both it is low: in the former, about one in 190; in the latter, about one in 280. Some of these were not immediately due to the operation, but to accidental complications. The immediate risk of the operation is not great; the remote risk as regards the causation of complications during pregnancy or parturition is also small, especially with the later methods of operating.

At the Geneva Congress it was agreed that all cases of retroversion complicated by adhesions were suitable for operation, and also all those associated with disease of the appendages.

Küstner, in performing Alexander's operation, sews the shortened ligament to the fascia between the upper end of the incision and the anterior superior spine. He records 120 cases of this (Alexander's) operation performed by others, and seventy-one performed by himself: of the 120, or rather of that

[1] *Loc. cit.* [2] *Samml. klin. Vortr.*, 1896, No. 171.

proportion of the total who reported themselves subsequently, there were thirteen failures, and pregnancy occurred in twenty-seven; five of these miscarried. Twenty labours were normal. His own results showed eight failures, or partial failures, out of sixty-five operations; pregnancy in five, all of which went to term, and were delivered normally.

Abbe[1] has described an important modification of Alexander's operation: after laying open the inguinal canal, freeing the ligament and drawing it out as far as necessary, he, by the use of a needle threaded with a loop of silk, uses the ligament as a suture to close the canal, thus gaining a firm attachment for it, and doing away with the necessity for either (1) tying the ligaments of opposite sides together, or (2) stitching the ligament to the surrounding tissues. He finds that this method does not impede the circulation in the ligament, and gives a very firm hold.

Bode[2] reports cases operated on by the vaginal route and intra-peritoneal method; his method follows that of Douglas, reefs being taken in the round ligaments near their origin from the uterus with silk sutures. The reefing near the origin of the ligament is of importance, in order that the tendency to approximation of the uterine cornua sometimes met with after operations of this kind may be avoided.

Wertheim and Mandl[3] combine two procedures: the vesico-uterine pouch is opened, and the cervix and fundus are drawn down so as to expose the tense utero-sacral ligaments. The intestines are held upward by a gauze compress, posterior adhesions are separated, and the sacro-uterine ligaments drawn backwards until the fundus is kept well forward; the points grasped are then stitched to the uterine origins of the utero-sacral folds, thus drawing backwards and upwards the isthmus and cervix; the round ligaments are then brought into view after removing the gauze packing and replacing the uterus, and shortened by the method of Bode previously described.

Pryor[4] opens the posterior *cul-de-sac* by a transverse incision, opening the peritoneum, the fingers are introduced, and any adhesions broken up. An iodoform gauze pad is inserted into the pelvis behind the cervix, with the patient in Trendelenburg's position, which causes the escape of the intestines into the abdominal cavity, and a second pad inserted, when the table is lowered to a horizontal position, the pads withdrawn, and a wad of gauze inserted just within the cut edges; the uterus is now brought into anteflexion bimanually, care being taken not to displace the gauze, which is removed after from seven to ten days under anæsthesia, and a fresh dressing applied; this is

1 *Ann. Surg.*, 1896, xxiv. 699.
2 *Centralbl. f. Gynäk.*, 1896, xx. 470; abstract in *Am. J. M. Sc.*, 1896, cxii. 621.
3 *Centralbl. f. Gynäk.*, 1896, xx. 465; abstract in *Am. J. M. Sc.*, 1896, cxii. 621.
4 *Med. Rec.*, 1895, xlviii. 76; quoted by Kelly, *loc. cit.*

allowed to remain a week, and a third dressing applied five days later. The vagina is kept packed for a month to support the uterus. A dense mass of lymph is produced about the utero-sacral ligaments, which draws the posterior pole of the uterus backwards and upwards.

Newman Dorland,[1] in discussing the subject of gestational complications and dystocia after anterior fixation of the uterus, said that shortening of the round ligaments in his opinion was very frequently unsuccessful, and that the objections to the opening of the peritoneal cavity having been overcome, ventrofixation was preferable; the objections that had lately arisen were based on the pernicious influence of the method on pregnancy and labour. He classified the methods and modifications of operations for reposition of the uterus into five groups: (1) shortening the round ligaments; (2) ventrofixation; (3) vaginal fixation; (4) vesical fixation; (5) cervico-fixation or trachelopexy. After all these operations, cases of dystocia and gestational troubles have been recorded.

He presents sixty cases of dystocia and gestational disturbance after ventrofixation alone. He presents 179 cases of pregnancy after ventrofixation, in 120 of which (67.03 per cent.) gestation was normal; in 111 (62.01 per cent.) labour was absolutely normal; in 68 (37.98 per cent.) both gestation and labour were absolutely normal. In 111 cases some disturbance of pregnancy, parturition, or both was noted.

In sixteen cases (8.93 per cent.) severe pain was felt in the line of the incision either during gestation or labour; in four cases excessive vomiting; in ten cases displacements of the cervix upwards and laterally; in nine cases rupture of the uterus was threatened; in five cases uterine inertia was noted; in fifteen abortions occurred; in ten miscarriages or premature labours; difficulties in parturition occurred in twenty-two cases; in nine abnormal presentations—ear once, breech twice, transverse six. Version was performed nine times; forceps, eleven times; Cæsarean section, three times; placenta retained, three times. Maternal deaths, two; fœtal deaths, 32. The great frequency of transverse presentations is due to the prevention of longitudinal expansion by the adhesions formed by the operation, which at the same time causes distension of the antero-posterior diameters, with thinning of the posterior wall. This can be avoided by the prevention of the formation of strong sero-fibrous adhesions. With this object Leopold favours the use of absorbable sutures; others remove the sutures about two weeks after operation.

The object of ventrofixation is not to convert a pathological retroversion into a pathological anteversion or flexion, but to restore and maintain the fundus uteri in its normal position of moderate anteflexion; the fundus should be fixed at such a

[1] *Univ. M. Mag.*, 1897, ix. 163; see also *Am. J. Obst.*, 1897, xxxv. 112.

height as not to cause undue upward traction on the cervico-vaginal attachments, or acute anteflexion or version.

Leopold advises fixation at a point not more than half an inch above the upper border of the symphysis. The lower the fixation the greater the proportion of disturbances of pregnancy and of cases of dystocia. Noble states that over twenty-five per cent. of pregnancies after the operations of Dührssen and Mackenrodt abort. Dührssen himself states that twenty-seven per cent. abort after vaginal fixation, while 16.6 of those treated for retrodisplacements by pessaries do so.

Dorland gives the following conclusions :—

1. From all points of view ventrofixation for the cure of uterine displacements gives the best result.

2. If improperly performed anterior fixation of the uterus may exert a deleterious effect upon subsequent pregnancies.

3. This evil increases in direct proportion to the low implantation of the fixation sutures, hence the vaginal operations are especially dangerous.

4. The abnormal conditions noted include pain and traction at the site of the incision, premature termination of the gestation, difficulties in micturition, excessive nausea and vomiting, abnormal presentations, mechanical obstruction to labour, uterine inertia, cervical rigidity, uterine rupture, placental anomalies, and *post-partum* and puerperal hemorrhages.

5. Over fifty per cent. of the subsequent gestations and labours offer some abnormal condition.

6. About fourteen per cent. suffer from abortions or miscarriages, and over twelve per cent. show dystocia, in varying degrees, after hysteropexy, while over twenty-seven per cent. abort after vaginal fixation.

Luigi Negri,[1] writing on hysteropexy studied from the obstetric point of view, gives noteworthy statistics of the influence of the various operations on pregnancy and parturition, and quotes Flaischlen and Veit, the former of whom says that ventrofixation has cured sterility in many cases of long standing, while the latter recommends the operation for some cases of sterility. The author finds twenty-five pregnancies in seventy-seven ventrofixations. Dührssen saw forty-four pregnancies in 318 vaginal fixations. Mackenrodt observed twelve pregnancies in forty-nine vaginal fixations, conception is therefore not only possible but frequent.

Among the cases noted were six of absolute sterility, which ceased in four after ventral, and in two after vaginal fixation. In twelve cases of sterility arising from displacements after labour of from two to eight years' duration, conception took place immediately after the operation; in four after ventro-fixation; in eight after vaginal fixation. In considering the behaviour of the adhesions created and the uterus thus fixed

[1] *Ann. di Ostet.* 1896, xviii. 584.

during pregnancy it is necessary to establish the fact of the existence of such fixation at the time of conception.

In 237 cases observed by Baudoin, the adhesions were present after three years; other observers have also noted their presence after long intervals.

As regards the behaviour of these adhesions during pregnancy and labour, the author quotes observations by numerous observers, which seem to indicate that the adhesions distend and stretch during pregnancy and after labour undergo involution. Sero-serous or sero-fibrous union is recommended according as the operators believe that stretching occurs or otherwise. Leopold, Laroyenne, and Sinclair make the adhesions as strong as possible. Some bury the sutures, others remove them after several weeks. The influence of the adhesions on the enlargement of the uterus in pregnancy and upon the course of labour is next noted. Negri collects 115 cases of pregnancy in ninety-seven women after ventrofixation; the result is known in 102: there were seventy-one labours at term, ten abortions took place, and four premature births; eight abortions and two premature labours were due to the operation. Five anomalous presentations: shoulder, three; foot, one; vertex with inclined parietals, one. Operative interference was required seventeen times. Cæsarean section, three times; forceps, seven; version (podalic), five; version (cephalic), one; extraction by the feet, one. In fourteen pregnancies there were disturbances due to the operation.

Norris,[1] writing on the management of labour complicated by ventrofixation, states that suspension of the uterus by a **technique** which avoids extensive and firm adhesions, and at the same time does not too sharply anteflex the uterus, and Alexander's operation of shortening the round ligaments are generally considered to be free from dangers to a subsequent pregnancy. He relates a case in which the labour terminating the first pregnancy after an operation for ventrofixation was attended with great difficulty and danger; the anterior wall fixed at the fundus by sutures had become folded on itself during its enlargement and formed a tumour obstructing delivery. No presenting part was felt through the cervix. A careful examination under ether showed that the posterior uterine wall, which practically formed the whole of the uterine sac, was so thinned that the intestine containing fæcal masses could be readily felt by the hand in the uterus. The fœtal head was lying high and on the left in the region of the mother's spleen. The breech lay in a depression between the upper margin of the mass of muscle (which represented the true anterior wall of the uterus) and the upper anterior uterine wall, with which the feet and legs were in contact. As it was impossible without imminent danger of rupturing the uterus to carry the hand past the obstruction and grasp the feet or dislodge the

[1] *Am. J. Obst.*, 1897, xxxv. 91, 116.

breech from the depression in which it rested, cephalic version was performed, and the head pressed down into the pelvis between the obstructing mass and the sacrum. Tarnier's axis traction forceps were then applied, and the child extracted; owing, however, to the umbilical cord having been compressed between the head and the mass of muscle above the symphysis, the child was dead. The puerperium was normal.

Her second pregnancy was terminated with difficulty at seven months, the foot presenting; the hypertrophied anterior wall, the rigid and thick anterior lip, and elevation of the cervix were present, but to a less degree than in the preceding pregnancy. The child was still-born.

From the statistics of Dorland and the cases described, the following suggestions are offered:—

Symptoms which suggest fixation of the uterus in an abnormal position are to be looked for as soon as pregnancy is recognised, such as excessive irritability of the bladder, dragging pain at the cicatrix, and excessive nausea and vomiting. Vaginal examination should be made to determine the degree of elevation of the cervix, the direction of the axis of its canal, the thickness of its anterior lip, and of the anterior uterine wall.

Careful bimanual examination should be repeated at intervals to ascertain the progress of these changes, and later in pregnancy abdominal palpation used to diagnose malpresentation.

The more nearly the cervical axis points above or towards the sacral promontory the greater will be the obstacle to delivery, which is also proportionate to the thickness of the anterior wall of the uterus and the anterior lip of the cervix; the chief difficulties arise from failure of dilatation owing to the imprisoning of the fibres of the anterior uterine wall. If these conditions are recognised the question of the advisability of inducing premature labour before the head is too large to obstruct delivery becomes a serious one.

Dorland's statistics show a higher fœtal mortality (fifty-eight per cent.) than in eclampsia, and a higher maternal mortality (fourteen per cent.) than in Cæsarean section.

The recognition of these conditions at full term calls for anticipation of faulty position, prolapse of cord, uterine inertia, and the dangers of rupture of the uterus. Efforts at dilatation, by manual or instrumental means, should be resorted to as soon as the cervix is sufficiently low in the pelvis. Should these fail, free incisions into the cervix should be resorted to. Serious obstruction must be dealt with without delay; the only alternatives are cervical dilatation or Cæsarean section, the latter is the indication in cases where the cervix is drawn up so high as to make manipulation and dilatation or incision difficult.

We may contrast the results of different operations for the cure of retro-displacements, the Continental and American tables being placed separately to avoid overlapping.

AMERICAN.

Kelly—219 operations, ventrofixation or suspension; fourteen pregnancies; difficult deliveries, one (forceps).

Edebohls—115 operations, shortening round ligaments; twelve pregnancies; two abortions; nine deliveries; seven living children; two lost sight of before delivery.

Dorland—179 cases of pregnancy after ventrofixation; sixty-eight normal labours; twenty-five abortions and miscarriages: twenty-two cases of difficult parturition; thirty-two fœtal deaths; two maternal deaths.

Norris—2 pregnancies after ventrofixation, both difficult; two fœtal deaths.

CONTINENTAL.

Negri—115 cases of pregnancy after ventrofixation; fourteen abortions and premature births; operative interference at parturition, seventeen.

Küstner—122 pregnancies after ventrofixation; fifteen abortions; six difficult deliveries.

Negri—112 pregnancies after vaginal fixation; nineteen abortions; nine difficult labours.

Küstner—23 pregnancies after vaginal fixation; thirteen abortions; five abnormal deliveries.

Küstner—27 pregnancies after Alexander's operation; five abortions; twenty normal labours.

Negri sums up the results as follows as regards abortions: interruption of pregnancy—(1) in untreated retroflexions, twenty per cent.; (2) in cases treated by pessaries, twelve per cent.; (3) after vaginal fixation, fifteen per cent.; (4) after ventrofixation, seven per cent.; (5) after shortening of round ligaments, four per cent.

WALTER C. SWAYNE.

